# The Use of Over-the-Counter (OTC) Skin-Lightening Agents and Their Side Effects Among the Residents of Saudi Arabia: A Cross-Sectional Study

**DOI:** 10.7759/cureus.62832

**Published:** 2024-06-21

**Authors:** Riyad M Abuhalimeh, Mona T Alanazi, Wafa T Alanazi, Mugrin R Alrwaili, Madhawi A Alanazi, Shuruq Mohsen A Alshammari

**Affiliations:** 1 Dermatology, Saudi Ministry of Health, Arar, SAU; 2 Medicine, Northern Border University, Arar, SAU

**Keywords:** observational cross-sectional study, public health and safety, saudi arabia, skin-lightening agents, over-the-counter

## Abstract

Introduction: Over-the-counter (OTC) skin-lightening agents are topical products in the form of lotions, creams, oils, soaps, and serums designed to alter skin pigmentation primarily for cosmetic purposes. The growing misuse and overuse of these products has become a serious public health concern due to their potential adverse effects on human health and their quality of life.

Methods: This study was cross-sectional in nature, adopting a convenience sampling technique utilizing data from a sample of 408 residents of Arar, Northern Saudi Arabia. The participants completed online questionnaires, distributed through social media means like Telegram, WhatsApp, and Facebook ensuring anonymity. Data was analyzed using SPSS Version 27 to obtain important insights.

Results: The sample of the study had a predominance of females 304 (74.5%) while only 104 (25.5%) were males. A substantial proportion of 170 (41.7%) of the participants were aged between 20 and 35 years with more than half (229 (56.2%)) being single and the majority (266 (65.2%)) having university and above level of education. More than half (259 (63.5%)) of them indicated that they had bought skin-lightening products without a doctor’s prescription. Twenty-eight (10.8%) of the participants had been diagnosed with a condition that increased skin pigmentation before with a substantial proportion (11 (39.3%)) noting that the product contained hydroquinone cream ingredients. The overall prevalence of OTC skin-lightening agents among the participants of Arar, Northern Saudi Arabia was 63.5% (259/408). The study found a significantly high prevalence of use of OTC skin-lightening agents among participants aged 20-35 years (87 (71.3%)) (p=0.031) as well as those who had a university level of education and above (129 (71.3%)) (p=0.001). The findings show that 39 (40.6%) of the female respondents and eight (30.8%) of the male participants experienced adverse effects associated with irritation and redness.

Conclusion: Overall, the study found a considerably high prevalence of the use of OTC skin-lightening agents among the participants of Arar, Northern Saudi Arabia. The use of OTC skin-lightening agents was significantly greater among female participants than male participants. The commonly used products contained hydroquinone cream ingredients, which presented adverse effects and complications associated with irritation, redness, and darkening of the skin. It is imperative to launch targeted public awareness campaigns to educate the community, especially women, about the risks associated with OTC skin-lightening agents, promote safer alternatives, and advocate for stricter regulation and control over the sale and distribution of skin-lightening products containing harmful ingredients.

## Introduction

The widespread use of over-the-counter (OTC) skin-lightening products has become commonplace globally [1]. This trend is influenced by societal norms, cultural inclinations, and individuals' aspirations for achieving an esthetically desirable appearance [1,2]. This phenomenon is not confined to specific regions or demographics; instead, global markets are inundated with a wide array of products promising lighter and brighter skin tones [3,4]. Saudi Arabia, like other countries, has witnessed an upsurge in the demand for such products [5]. This delves into the use and side effects associated with the use and misuse of OTC skin-lightening agents in the Northern Border Region of Saudi Arabia, aiming to address the knowledge gap regarding this growing public health concern.

Skin-lightening products come in various forms such as creams, lotions, soaps, and serums, and are designed to alter skin pigmentation by reducing melanin production [2]. Melanin, the primary determinant of skin color, plays a crucial role in protecting the skin against harmful ultraviolet (UV) radiation [2]. Different sociocultural factors have fostered a preference for lighter skin tones in certain communities, leading to the widespread use of these products despite potential health risks [6]. Hydroquinone, a tyrosinase inhibitor, is a prominent active ingredient in many skin-lightening products [7,8]. By interfering with melanin synthesis, hydroquinone can reduce hyperpigmentation and even out skin tone. Yet, its safety profile has raised concerns. High concentrations of hydroquinone have been associated with skin irritation, contact dermatitis, and paradoxical hyperpigmentation, particularly in individuals with darker skin tones. Furthermore, there are concerns about its potential to cause ochronosis, a skin condition characterized by blue-black discoloration and thickening of the skin [7,8]. Apart from hydroquinone, corticosteroids and retinoids are also utilized in skin-lightening formulations for their anti-inflammatory and exfoliating properties, respectively. However, their misuse and overuse can lead to a range of adverse effects. Prolonged use of potent corticosteroids can cause skin thinning, telangiectasia, and even systemic absorption, leading to Cushing's syndrome or adrenal suppression. Retinoids, on the other hand, are associated with skin irritation, redness, and increased sensitivity to UV radiation [9,10]. A significant concern lies in the unregulated and counterfeit skin-lightening products that flood markets [11]. These products may contain undisclosed and potentially harmful ingredients, including heavy metals such as mercury and lead. Prolonged exposure to these toxic substances can lead to systemic effects, including neurological and nephrological disorders. Furthermore, some products may contain allergens or irritants that can exacerbate existing skin conditions or induce new ones [11].

Promoting lighter skin tones as a beauty ideal carries significant ethical and societal implications. It reinforces detrimental beauty standards, perpetuates colorism, and amplifies feelings of inferiority among individuals with darker skin tones. This can affect not only the physical appearance aspect but can have far-reaching psychological effects, impacting self-esteem and mental well-being [6].

The OTC skin-lightening agents are a complex issue that demands a multifaceted approach. Several regulatory agencies struggle to monitor and control these products effectively. In Saudi Arabia, the Saudi Food and Drug Authority (SFDA) oversees the regulation of cosmetic products, yet gaps in enforcement and consumer education persist. Understanding the extent of their usage can provide insights into the community's perceptions of beauty and skincare practices. Assessing the prevalence of side effects is crucial for public health interventions, as these products may contain ingredients that pose health risks or exacerbate skin conditions.

## Materials and methods

Study design

This study employed a descriptive cross-sectional design. The study was conducted between the period of October 2023 and March 2024 and involved the adult population in Arar City, Northern Saudi Arabia.

Inclusion and exclusion criteria

The study encompassed individuals of both genders aged 18 years and above residing in Arar City. It included participants who did not exhibit any chronic skin lesions and who demonstrated a willingness to engage in the study procedures.

The study excluded individuals under the age of 18, residents outside of Arar City, chronic skin lesions with persistent and long-lasting abnormalities, and individuals who declined to participate in the study.

Sampling technique and sample size

This study adopted a convenient sampling technique, and participants were enrolled in the study based on their availability and willingness to participate during the time of data collection. To evaluate the sample size, we used the Cochrane sample size approach, n = Z2(1 − p)/d2; where n is the sample size, Z denotes the critical statistic for a 95% confidence interval, the anticipated prevalence was set at 50%, and d is the margin of error, set at 0.05. According to our calculations, the minimum acceptable sample size was determined to be 384 respondents. However, in order to ensure the robustness and reliability of our findings, we opted for a larger sample size of 408 respondents.

Data collection tools and procedures

The researchers crafted the questionnaire-based aims and objectives. To ensure the questionnaire's reliability, 10 respondents participated in a pilot phase aimed at evaluating its feasibility, clarity, and understandability. To confirm that participants were truly residing in Arar, the questionnaire included a mandatory question asking for the participant's current place of residence, which had to be Arar for inclusion in the study. The respondents involved in the pilot study were not included in the main study. Following adjustments based on pilot feedback, the questionnaire was distributed online to respondents via WhatsApp and Telegram groups. The questionnaire used in this study was carefully designed to gather comprehensive data across several sections. It included socio-demographic variables such as age, gender, and education level to understand the background of the participants. The section on OTC skin-lightening agents encompassed questions about the source of information, potential active ingredients, and reasons for use, along with specific products used. Additionally, the questionnaire detailed the complications section, where participants reported any adverse effects experienced using a combination of multiple-choice and open-ended questions to capture both quantitative and qualitative data.

Data analysis

After the data collection exercise, the data underwent a thorough data cleaning exercise. The data cleaning process involved steps like removing duplicate entries, scrutinizing for outliers, and addressing any missing data points through methods such as imputation or exclusion, depending on the extent and impact of the missing information. After data cleaning, data was coded and entered in Sciences (SPSS) software version 27.0 for analysis. SPSS was used because it has a wide range of statistical tests and procedures. Categorical variables were presented in terms of count and frequencies and the Chi-square test was used to determine association between categorical variables with a predetermined significance level set at p<0.05. The Chi-square test was employed to analyze the data because it is well-suited for determining associations between categorical variables.

Ethical considerations

For this study, ethical approval was obtained from the Institutional Review Board in ArAr (NB-IRB-023-10-040). Important ethical considerations were carefully addressed throughout the study, notably focusing on upholding confidentiality and ensuring informed consent. Before participating, all individuals were provided with comprehensive information regarding the study's objectives, risks, and benefits. Participants had to provide voluntary and informed consent without coercion. The researchers reassured the respondents of their privacy by protecting their identity and sensitive information.

## Results

Table 1 shows 408 participants from Arar, Northern Saudi Arabia took part in the study. More than half (304 (74.5%)) of the participants were females while only 104 (25.5%) were males. A substantial proportion of 170 (41.7%) of the participants were aged between 20 and 35 years with more than half (229 (56.2%)) being single and the majority (266 (65.2%)) of them having a university and above level of education. Furthermore, a considerable proportion (159 (39.0%)) were working with most of them (142 (34.8%)) having good monthly income of the family.

**Table 1:** Socio-demographic information of the participants (N=408) Socio-demographic information presented in frequencies (n) and proportion (%).

Demographic information	Category	Frequency and proportion, n (%)
Sex	Female	304 (74.5%)
	Male	104 (25.5%)
Age (years)	18-20	123 (30.1%)
	20-35	170 (41.7%)
	35-40	45 (11.0%)
	40-45	50 (12.3%)
	50-55	18 (4.4%)
	60 or more	2 (0.5%)
Marital status	Married	156 (38.2%)
	Single	229 (56.2%)
	Widow	3 (0.7%)
	Divorced	20 (4.9%)
Educational level	Uneducated	7 (1.7%)
	Primary	3 (0.7%)
	Intermediate	19 (4.7%)
	High school	113 (27.7%)
	University and above	266 (65.2%)
Working condition	Working	159 (39.0%)
	Unemployed	91 (22.3%)
	Full-time student	158 (38.7%)
Monthly income of the family	Weak	25 (6.1%)
	Middle	123 (30.2%)
	Good	142 (34.8%)
	Excellent	118 (28.9%)

Table 2 depicts information about the OTC skin-lightening agents use, products, and their complications among the participants. The vast majority (378 (92.6%)) of the participants had accounts on social media platform sites (TikTok, Facebook, Twitter, etc). More than half (259 (63.5%)) of them indicated that they had bought skin-lightening products OTC; having no doctor’s prescription. Of them, nearly half (128 (49.4%)) were recommended the products by their family members and friends. About half (134 (51.7%)) of the participants indicated that they bought the products from pharmacies without a doctor’s prescription. Twenty-eight (10.8%) of the participants had been diagnosed with a chronic skin pigmentation condition before with a substantial proportion (11 (39.3%)) noting that the product contained hydroquinone cream ingredients. The study noted that more than half (186 (71.8%)) of the participants reported having ever used sunscreen cream at the time of the survey with (93 (35.9%)) of them having used the products for two to four weeks. More than half (152 (58.7%)) of the participants indicated that there were favorable results in using the products without a doctor’s prescription. The majority (137 (52.9%)) of the participants did not report any complications with some reporting complications such as severe irritation and redness (47 (18.1%)) and darkening of the skin (37 (14.3%)). The majority of the participants (156 (60.2%)) would recommend the products to their friends and family members.

**Table 2:** OTC skin-lightening agents use, products, and their complications among the participants OTC skin-lightening agents use, products, and their complications presented in frequencies (n) and proportion (%). OTC, over-the-counter

Statements	Categories	Frequency and proportion, n (%)
Do you have an account on social networking sites (TikTok, Facebook, Twitter, etc.)?	Yes	378 (92.6%)
	No	30 (7.4%)
Have you ever bought skin-lightening products without a doctor’s prescription?	Yes	259 (63.5%)
	No	149 (36.5%)
Did you get these products based on	Recommendations from family and friends	128 (49.4%)
	TV ads	15 (5.8%)
	Social media sites	116 (44.8%)
The average amount of money you spend on skin-lightening products without a doctor’s prescription based on your monthly income?	Few	96 (37.1%)
	Medium	130 (50.2%)
	A lot	33 (12.7%)
Do you think more expensive medication/products help lighten dark/brown spots more?	Yes	90 (34.7%)
	No	69 (26.6%)
	Maybe	100 (38.7%)
Where to get skin-lightening products without a doctor’s prescription?	Pharmacies	134 (51.7%)
	Cosmetic offices	33 (12.7%)
	Buy online	80 (30.9%)
	Friends	11 (4.2%)
	Others	1 (0.4%)
Have you been diagnosed with a disease that causes increased skin pigmentation before? If yes, state the diagnosis	Yes	28 (10.8%)
	No	231 (89.2%)
What is the active ingredient in these products?	Tretinoin cream	7 (25.0%)
	Azelaic acid cream	6 (21.4%)
	Hydroquinone cream	11 (39.3%)
	Retinol cream	1 (3.6%)
	Mometasone cream	3 (10.7%)
Do you use sunscreen cream?	Yes	186 (71.8%)
	No	73 (28.2%)
Is there result of using skin-lightening products without doctor’s prescription?	Yes	142 (54.8%)
	No	28 (10.8%)
	Maybe	89 (34.4%)
How long have you been using these products?	2-4 weeks	93 (35.9%)
	5-8 weeks	41 (15.8%)
	9-12 weeks	21 (8.2%)
	Less than two weeks	62 (23.9%)
	More than 12 weeks	42 (16.2%)
Is the result favorable or unfavorable?	Favorable	152 (58.7%)
	Unfavorable	36 (13.9%)
	I don’t know	71 (27.4%)
Do these products cause any of these complications?	Darkening of the skin	37 (14.3%)
	Irritation and redness	47 (18.1%)
	Burns	7 (2.7%)
	Pills	24 (9.3%)
	White spots	2 (0.8%)
	The appearance of capillaries under the skin	5 (1.9%)
	No complication	137 (52.9%)
Do you recommend such products to your friends or relatives?	Yes	156 (60.2%)
	No	103 (39.8%)

According to the study results, the overall prevalence of use of OTC skin-lightening agents among the participants of Arar, Northern Saudi Arabia was 63.5% (259/408).

Table 3 presents the prevalence of the use of OTC skin-lightening agents based on the participants’ socio-demographic characteristics. The study found the prevalence of the use of OTC skin-lightening agents to be significantly greater among female participants (209 (80.7%)) than male participants (50 (19.3%)) (p=0.002).

**Table 3:** Prevalence of use of OTC skin-lightening agents by socio-demographic data of the participants Association between socio-demographics information and prevalence of use of OTC skin-lightening agents among the participants. *Significant at p<0.05 level OTC, over-the-counter

Socio-demographic characteristics		Use of OTC skin-lightening agents				P-value
		Low usage		High usage		
		n	%	n	%	
Sex	Female	45	21.5%	164	78.5%	0.002*
	Male	43	86.0%	7	14.0%	
Age (years)	18-20	26	45.6%	31	54.4%	0.031*
	20-35	35	28.7%	87	71.3%	
	35-40	15	45.5%	18	54.5%	
	40-45	18	52.9%	16	47.1%	
	50-55	7	58.3%	5	41.7%	
	60 or more	1	100%	0	0	
Marital status	Married	69	59.5%	47	40.5%	0.312
	Single	63	48.1%	68	51.9%	
	Widow	1	100%	0	0	
	Divorced	6	54.5%	5	45.5%	
Educational level	Uneducated	4	66.7%	2	33.3%	0.001*
	Primary	2	66.7%	1	33.3%	
	Intermediate	7	53.8%	6	46.2%	
	High school	31	55.4%	25	44.6%	
	University and above	52	28.7%	129	71.3%	
Working condition	Work	49	43.4%	64	56.6%	0.23
	I do not work	38	59.4%	26	40.6%	
	Student	34	41.5%	48	58.5%	
Monthly income of the family	Weak	11	73.3%	4	26.7%	0.003*
	Middle	44	53.0%	39	47.0%	
	Good	41	46.6%	47	53.4%	
	Excellent	27	37.0%	46	63.0%	

The study found a significantly high prevalence of use of OTC skin-lightening agents among participants aged 20-35 years (87 (71.3%)) (p=0.031) as well as those who had a university level of education and above (129 (71.3%) (p=0.001)). Furthermore, a statistically significant high prevalence of use of OTC skin-lightening agents was discovered among participants whose families had good and excellent monthly incomes (p=0.003)

Table 4 depicts the adverse effects of the use of OTC skin-lightening agents. The findings show that 39 (40.6%) of the female respondents and eight (30.8%) of the male participants experienced adverse effects associated with irritation and redness. The adverse effects were found to be higher among females than males. 96 (78.7%) of the female participants and 26 (21.3%) of the male participants showed varying adverse effects occasioned by the use of OTC skin-lightening agents. There was a statistically significant difference in the adverse effects of OTC skin-lightening agents between female and male participants (p=0.014).

**Table 4:** Adverse effects of OTC skin-lightening agents in relation to gender *Based on the Chi-squared test OTC, over-the-counter

	Complications						
Sex	Darkening of the skin, n (%)	Irritation and redness, n (%)	Burn, n (%)	Pills, n (%)	White spots, n (%)	The appearance of capillaries under the skin, n (%)	P-value
Male	5 (19.2%)	8 (30.8%)	2 (7.7%)	8 (30.8%)	1 (3.8%)	2 (7.7%)	0.014*
Female	32 (33.3%)	39 (40.6%)	5 (5.2%)	16 (16.7%)	1 (1.0%)	3 (3.1%)	

## Discussion

The study sought to determine the prevalence of the use of OTC skin-lightening agents among the participants of Arar, Northern Saudi Arabia. The study sample was primarily made up of females, aged between 20 and 35 years, and singles with the majority with a university and above level of education.

The findings found the prevalence of the use of OTC skin-lightening agents to be 63.5% (259/408) among the participants. The results of the current study showed a higher prevalence of the use of skin-lightening products compared with the study conducted by Bamerdah et al., which found a prevalence of 35.7% in the general population of Western Saudi Arabia [12]. The disparity in prevalence rate between the two studies could be a result of differences in sample size and the gender distribution of the participants across the studies. The study found the prevalence of the use of OTC skin-lightening agents to be significantly greater among female participants than male participants.

The study established a significantly high prevalence of use of OTC skin-lightening agents among participants aged 20-35 years as well as those who had a university level of education and above. The findings are consistent with those of the study conducted in Ghana by Lartey et al., which found that participants aged below 40 years were more likely to use OTC skin-lightening products than those aged above that age [13]. However, the study conducted in Northern Saudi Arabia by Alrayyes et al. found no significant difference between women who used OTC skin-lightening cosmetics and those who did not use them based on their age groups; this observation could be informed by strong cultural and religious bias against the use of skin-lightening agents across different age groups in Saudi Arabia [14]. Regarding the education level, the findings of the current study are similar to those of the study by Al-Saleh, 2016, which reported significant differences in education levels with college-educated participants indicating a high prevalence of the use of skin-lightening cosmetics, which could be due to increased exposure to the agents in colleges and influence from other students of diverse cultures [15]. Additionally, a statistically significant high prevalence of use of OTC skin-lightening agents was discovered among participants whose families had good and excellent monthly incomes.

The study revealed that nearly half of the participants bought the products from pharmacies without a doctor’s prescription. This is similar to the study conducted in Jordan by Hamed et al., which reported that the majority of the users of skin-lightening products bought them from pharmacies or cosmetic skin care shops [16]. According to the findings, a substantial proportion of the participants reported that the products they used contained hydroquinone cream ingredients with some participants reporting complications such as irritation and redness as well as darkening of the skin. Comparably, the study by Bamidele et al. found that hydroquinone was the commonly used skin-lightening agent though it was characterized by side effects such as contact dermatitis, irritant dermatitis, and ochronosis, which had the potential of causing skin discoloring on prolonged exposure [17]. The study noted that more than half of the participants used sunscreen cream with a considerable proportion having used the products for two to four weeks. The findings concur with those of the study by Dlova et al. in South Africa where more than half of the South African women surveyed reported having used sunscreen creams for many years [18].

The adverse effects were found to be higher among females than males. A statistically significant difference in the adverse effects of OTC skin-lightening agents was established between female and male participants. The study noted that the majority of the female and male participants experienced adverse effects from the use of skin-lightening agents associated with irritation and redness. The findings are similar to those of Jha et al., which reported systemic and immediate side effects of OTC skin-lightening agents associated with stinging and irritation [19].

The major restriction and limitation of the study was the utilization of a cross-sectional study design, which can only identify relationships between components but not causalities. As this was a survey-based study, recollection bias might be a limitation that also needs further investigation. Furthermore, because the study involved only one region in Saudi Arabia, its conclusions cannot be applied to all Saudi Arabian regions.

## Conclusions

Overall, the study found a considerably high prevalence of the use of OTC skin-lightening agents among the participants of Arar, Northern Saudi Arabia. The use of OTC skin-lightening agents was significantly greater among female participants than male participants. The commonly used products contained hydroquinone cream ingredients, which presented adverse effects and complications associated with irritation, redness, and darkening of the skin. While the study results might be limited by sampling method biases and potential confounding variables, the high prevalence of use of OTC skin-lightening agents noted in the current study calls for public awareness campaigns about the potential risks and adverse effects related to the misuse of OTC skin-lightening agents.
